# COVID-19 clinical and laboratory diagnosis overview

**DOI:** 10.1186/s42506-021-00087-w

**Published:** 2021-08-18

**Authors:** Rania A. Zayed, Dalia Omran, Abeer A. Zayed

**Affiliations:** 1 Clinical and Chemical Pathology Department, Faculty of Medicine, Cairo University, Cairo, Egypt; 2 Department of Endemic Medicine and Hepatogastroentrology, Faculty of Medicine, Cairo University, Cairo, Egypt; 3 Department of Forensic Medicine and Toxicology, Faculty of Medicine, Cairo University, Cairo, Egypt

**Keywords:** COVID-19, Serology, RT-PCR, Lab, Clinical

## Abstract

**Background:**

COVID-19 was identified in Wuhan, China, in December 2019, and rapidly spread worldwide, being declared global pandemic on the 11th of March 2020. Since its emergence, COVID-19 has raised global concerns associated with drastic measures that were never adopted in any previous outbreak, to contain the situation as early as possible.

**Main body:**

The 2019 novel corona virus (2019-nCoV) or SARS-CoV-2 is the causative agent of COVID-19. 2019-nCoV genetic sequence was rapidly identified within few days since the first reported cases and RT-PCR kits became available for COVID-19 diagnosis. However, RT-PCR diagnosis carries a risk of false-negative results; therefore, additional serologic tests are needed. In this review, we summarize the clinical scenario that raises suspicion of COVID-19 and available laboratory diagnostics.

**Conclusion:**

The most important approach in the battle against COVID-19 is rapid diagnosis of suspicious cases, timely therapeutic intervention and isolation to avoid community spread. Diagnosis depends mainly on PCR testing and serological tests. However, even in the context of negative lab test results and clinical suspicion of COVID-19 infection, clinical decision should be based on clinical suspicion.

## Background

The 2019 novel corona virus (2019-nCoV)/SARS-CoV-2 sequence was first identified in January 2020 from bronchoalveolar lavage (BAL) samples of five patients in Wuhan, China, presenting with unusual respiratory symptoms; fever, cough, and dyspnea accompanied by complications of acute respiratory distress syndrome with diffuse lung opacities and consolidation detected in chest radiography. Next generation sequencing results revealed an unknown β-CoV strain with 79.0% nucleotide identity with the sequence of SARS-CoV, 51.8% identity with the sequence of MERS-CoV and 87.7% nucleotide identity with bat SARS-like CoV ZC45 [1]. Therefore, it was announced that the 2019-nCoV is of bat origin. In fact, bats are the key reservoir of CoVs, and many human CoVs most probably have originated from bats [2, 3].

The disease caused by 2019-nCoV/SARS-CoV-2 was named as coronavirus disease 2019 (COVID-19) by the World Health Organization (WHO) [4]. On 30 January 2020, COVID-19 was declared by the WHO as a public health emergency of international concern (PHEIC) [5]. In 2005, the WHO gained the power to declare an international emergency [6], since then, international emergency was declared five times: H1N1 swine flu in 2009, the Ebola outbreak in West Africa in 2013, the polio outbreak in 2014, the Zika outbreak in 2016, and Ebola outbreak in the Democratic Republic of Congo in 2019 [6]. However, none of these previous emergencies has led to a worldwide pandemic [7]. Because of the rapid increase in numbers of COVID-19 cases and uncontrolled worldwide spread, it was declared by the WHO a pandemic on 11th of March 2020 [8]. As of July 16, 2020, the virus has infected 13,378,853 total confirmed cases with 580,045 deaths [9]. COVID-19 pandemic was associated with strict measures to contain the situation where many countries closed their borders associated with partial lockdown of most daily activities and social distancing. The incubation period of COVID-19 is believed to be as long as 14 days, with potential asymptomatic transmission [10, 11]. COVID-19 is highly contagious and has higher transmissibility (R0, 1.4–5.5) than both SARS-CoV (R0, 2–5) and MERS-CoV (R0, < 1) [12], although the mortality rate is lower 3.4% for COVID-19, compared to 10% for SARS-CoV and 34% for MERS-CoV [13–15].

## COVID-19 diagnosis

### Clinical presentation

The China National Health Commission proposed guidelines for initial diagnosis and disease severity triage into mild, severe, and critical categories. Around 70 to 80% of patients are mild, and 20 to 30% are severe or critical [16] (Table 1).

**Table 1:** COVID-19 severity triage

Mild	Severe	Critical
• Fever • Respiratory symptoms • Pneumonia on chest radiography	• **Need to meet one of the following criteria:** • Respiratory distress, RR ≥ 30/min • Resting blood oxygen saturation ≤ 93% • Arterial blood oxygen partial pressure (PaO2)/FiO2 ≤ 300 mmHg	• **Need to meet one of the following criteria:** • Respiratory failure needing mechanical oxygenation • Shock • Development of other organ failures, requiring intensive care unit care

Clinical diagnosis requires epidemic exposure, in addition to two clinical findings of the following: fever, radiographic features, normal or lowered white blood cells, or reduced lymphocyte count [16].

### Real-time-PCR

The current diagnostic test for COVID-19 is RT-PCR assay [17]. It would not be possible to do PCR test to all suspected individuals, so the Centers for Disease Control and Prevention (CDC) released guidance for priorities for COVID-19 PCR testing [18] (Table 2).

**Table 2:** Priorities for PCR testing of suspected COVID-19 patients according to CDC guidelines [18]

Priority 1 ***Ensure optimal care options for all hospitalized patients, lessen the risk of nosocomial infections, and maintain the integrity of the healthcare system***	• Hospitalized patients • Symptomatic healthcare workers
Priority 2 ***Ensure that those who are at highest risk of complication of infection are rapidly identified and appropriately triaged***	• Patients in long-term care facilities with symptoms • Patients 65 years of age and older with symptoms • Patients with underlying conditions with symptoms • First responders with symptoms
Priority 3 ***As resources allow, test individuals in the surrounding community of rapidly increasing hospital cases to decrease community spread, and ensure health of essential workers***	• Critical infrastructure workers with symptoms • Individuals who do not meet any of the above categories with symptoms • Healthcare workers and first responders • Individuals with mild symptoms in communities experiencing high 2019-nCoV hospitalizations
Non-priority	• Individuals without symptoms

The recommended specimen for testing is lower respiratory tract specimen: sputum and/or endotracheal aspirate or bronchoalveolar. If not possible or in asymptomatic contacts, upper respiratory tract specimen, nasopharyngeal and oropharyngeal swab, or wash in ambulatory patients is collected, with preference of combined nasopharyngeal swab and oropharyngeal swab collection [19].

High viral loads in both upper and lower respiratory tract are detected 5-6 days of the onset of symptoms [20–23]. Lower respiratory tract specimens yield highest viral loads for the diagnosis of COVID-19 [24]. As for upper respiratory tract specimens higher sensitivity of nasopharyngeal swabs (63%) was detected compared to oropharyngeal swabs (32%) [25].

Available RT-PCR testing targets two genes in the virus genome: the E and RdRP genes. The E assay is specific for all SARS-CoV-related viruses, while the RdRP assay only detects the COVID-19 virus, the recommendation for laboratory confirmation of cases is to detect two different genetic targets: E followed by RdRP [26]. However, in areas where COVID-19 virus is widely spread, positive RT-PCR test result requires detection of at least one target gene, with priority to the E gene being more sensitive [26]. It should be well clear that one or more negative results do not rule out the possibility of COVID-19 virus infection, as false negative result in an infected individual may be caused by several factors: (1) poor quality of the specimen; (2) timing of specimen collection, late or very early in the infection; (3) inappropriate specimen handling and/or and shipping; and (4) technical error in the test. It is recommended that if a negative result is obtained from a patient with a high index of suspicion for COVID-19 virus infection, particularly when only upper respiratory tract specimens were collected, additional specimens should be collected and tested preferably from the lower respiratory tract [27]. Rectal swab can be used in suspicious patients and close contacts with confirmed cases, who test negative for COVID-19 in throat swab as some patients were found to have viral RNA in their feces starting from 1 day after infection and for up to 12 days [28].

### Serological tests

Serological testing detects antigens and antibodies directed against the virus. SARS-CoV2 belongs to the same family of β-coronaviruses as those caused SARS and MERS outbreaks; it is expected to have similar antibody generation process [29] where there is a lag period of 14-28 days after the onset of illness till the antibodies appears in patients’ serum [30]. In some people with COVID-19 disease confirmed by RT-PCR, weak, late, or absent antibody responses have been reported [31, 32]. The strength of antibody response is dependent on multiple factors as age, nutritional status, disease severity, and certain medications or infections that may suppress the immune system [31–33].

In March 2020, the FDA issued a policy that allows developers of certain serological tests to begin to market or use their tests once appropriate evaluation to ensure test validation is performed. The FDA issued this policy to allow early patient access to certain serological tests. Until 17 July 2020, thirty serology/antibody tests and two antigen diagnostic tests for SARS-CoV-2 were issued an Emergency Use Authorization (EUA) intended for use by clinical laboratories [34]. Criteria for EUA were as follows: (1) The SARS-CoV-2 can cause a serious or life-threatening disease or condition, including severe respiratory illness, to humans infected by this virus; (2) based on the totality of scientific evidence available to FDA, it is reasonable to believe that the product may be effective in diagnosing COVID-19, and that the known and potential benefits of the product when used for diagnosing COVID-19 outweigh its known and potential risks; and (3) there is no adequate, approved, and available alternative to the emergency use of the product [35].

It is recommended to use combined IgG and IgM antibody testing for more accurate results [29]. The average time for seroconversion in reported studies is 12 days, while positive RT-PCR is detected 5-6 days from the onset of symptoms, making antibody testing still inferior to RT-PCR in COVID-19 diagnosis but more likely used when RT-PCR is not available or accessible [36].

Also, cross-reactivity of other respiratory viruses with SARS-CoV-2 is reported and may influence serology test results, especially in patients recently exposed to respiratory infections [37].

A systematic review including 55 publications analyzing 8526 SARS-CoV-2 patients’ samples from Asia (*n* = 38), Europe (*n* = 15), USA (*n* = 1), and China (*n* = 1), concluded that results for IgG, IgM, IgA, total antibodies, and IgG/IgM, all showed low sensitivity during the first week from onset of symptoms (less than 30.1%), rose in the second week and reached their highest values in the third week. The combination of IgG/IgM had a sensitivity of 30.1% (95% CI 21.4 to 40.7) for 1 to 7 days, 72.2% (95% CI 63.5 to 79.5) for 8 to 14 days, 91.4% (95% CI 87.0 to 94.4) for 15 to 21 days. Therefore, antibody testing is not recommended as primary tool in COVID-19 diagnosis but provides complimentary diagnostic tool in RT-PCR test—negative patients presenting late or in detecting past infection when done 15 days or more after the onset of symptoms [38].

A study by Carmen et al. evaluated six different commercial enzyme immunoassays (EIA) platforms and eight points of care tests (POCT) with the same serum panel to identify the sensitivity of the available assay in COVID-19 diagnosis over time ranging from as early as 3 days post symptoms onset to > 45 days post symptoms onset. Their conclusion was that serology assays should not be used for the diagnosis of acute infections [39].

The American Society for Microbiology (ASM) [40], the WHO [41], the Centers for Disease Control and Prevention [42], and the Public Health Agency of Canada [43] also published similar recommendations against using serology testing for diagnosis of acute infection.

## Conclusion

COVID-19 available diagnostics puts the health authorities in challenging situation as diagnosis based on clinical symptoms alone is inaccurate, in addition to the presence of asymptomatic carriers and long incubation period of the virus. False negative RT-PCR results in infected patients adds to the challenge, necessitating the need for a rapid and sensitive technique to be available in most laboratories for swift detection of COVID-19 in order to limit spread and properly treat infected individuals. Available diagnostic tests alone are not enough to provide guaranteed diagnosis of COVID-19. Clinical suspicion of COVID-19 should be thoroughly taken in consideration even with negative test results to allow timely management of COVID-19, contain and limit the damage of current outbreak.

To monitor disease progression, it is recommended to combine both serial viral load monitoring and antibody response, as viral load was found to be inversely related to serum antibody response [22].

Detection of antibody responses to COVID-19 in the population is important to aid vaccine development, and to add to our understanding of the extent of infection among individuals who passed asymptomatic and/or not identified during surveillance efforts. It has to be noted that positive antibody testing does not guarantee safety from reinfection by COVID-19 or acquisition of herd immunity, and therefore should not be considered as an excuse to ignore public health advice which may lead to increasing the risk of continued transmission.

## Data Availability

Not applicable.

## Abbreviations

RT-PCR: Real-time-PCR
β-CoV: β-coronaviruses
SARS-CoV: Severe acute respiratory syndrome-related coronavirus
MERS-CoV: Middle East respiratory syndrome coronavirus
CoVs: Coronaviruses
RR: Respiratory rate
E gene: Envelope protein gene
RdRP gene: RNA-dependent RNA polymerase gene
Ig: Immunoglobulin
